# Desirable attributes of theories, models, and frameworks for implementation strategy design in healthcare: a scoping review protocol

**DOI:** 10.12688/f1000research.124821.1

**Published:** 2022-09-06

**Authors:** Joshua Porat-Dahlerbruch, Guillaume Fontaine, Ève Bourbeau-Allard, Anne Spinewine, Jeremy M. Grimshaw, Moriah E. Ellen

**Affiliations:** 1 Israel Implementation Science and Policy Engagement Centre, Ben-Gurion University of the Negev, Be'er Sheva, 8410501, Israel; 2 Department of Health Policy and Management, Ben-Gurion University of the Negev, Be'er Sheva, 8410501, Israel; 3 U.S.-Israel Fulbright Commission, Tel Aviv, Israel; 4 Centre for Implementation Research, Clinical Epidemiology Program, Ottawa Hospital Research Institute, Ottawa, Ontario, Canada; 5 Centre for Nursing Research, Jewish General Hospital, CIUSSS West-Central Montreal, Quebec, Canada; 6 Faculty of Medicine, University of Ottawa, Ottawa, Ontario, Canada; 7 Clinical Pharmacy Research Group, Louvain Drug Research Institute,, Université catholique de Louvain, Louvain, Belgium; 8 Department of Pharmacy, CHU UCL Namur, Namur, Belgium

**Keywords:** implementation; implementation science; review; theory; evidence-based practice; literature review; protocol; knowledge translation

## Abstract

**Background: **Implementation strategies can facilitate the adoption of evidence-based practices and policies. A wide range of theoretical approaches—theories, models, and frameworks—can be used to inform implementation strategy design in different ways (e.g., guiding barrier and enabler assessment to implementing evidence-based interventions). While selection criteria and attributes of theoretical approaches for use in implementation strategy design have been studied, they have never been synthesized. Furthermore, theoretical approaches have never been classified according to desirable criteria and attributes for use in implementation strategy design. This scoping review aims to a) identify the literature reporting on the selection of theoretical approaches for informing implementation strategy design in healthcare and b) understand the suggested use of these approaches in implementation strategy design.

**Methods: **The Joanna Briggs Institute methodological guidelines will be used to conduct this scoping review. A search of three bibliographical databases (MEDLINE, Embase, CINAHL) will be conducted for peer-reviewed discussion, methods, protocol, or review papers. Data will be managed using the Covidence software. Two review team members will independently perform screening, full text review and data extraction.

**Results:** Results will include a list of selection criteria and attributes of theoretical approaches for use in research on implementation strategy design. Descriptive data regarding selection criteria and attributes will be synthesized graphically and in table format. Data regarding the suggested use of theoretical approaches in implementation strategy design will be presented narratively.

**Conclusions: **Results will be used to classify existing theoretical approaches according to the attributes and selection criteria identified in this scoping review. Envisioned next steps include an online tool that will be created to assist researchers in selecting theories, models, and frameworks for implementation strategy design.

## Background

Implementation strategies are methods or techniques used to facilitate the adoption of evidence-based interventions by healthcare providers, organizations and systems. ^1^^,^^2^ These strategies must address the specific implementation context. ^2^^,^^3^ Rigorous, systematic and context-specific design of implementation strategies is critical for informing the implementation of evidence-based interventions. ^4^^–^^6^ Implementation strategy design is often guided by *informal* (or implicit) theory—i.e., an “understanding of the problem and its determinants gained through experience or tacit knowledge by the developers of the intervention.” ^4^^–^^6^ While informal theory can be useful for developing strategies suited to the particular implementation context, there are downsides to using only informal theory in this context. These include lack of standardization of language/tools, failure to build on existing knowledge, and influence of personal preferences/biases. ^7^ To overcome these limitations and inform implementation strategy design, implementation scientists and practitioners can select from a broad range of theories, models, and frameworks (TMFs), which can be classified as *formal* theory. ^8^^,^^9^

Several methodological approaches can be used to support the application of formal theory to the design of implementation strategies. ^2^^,^^10^ Typically, these approaches involve identifying which stakeholders need to do what differently, identifying barriers and enablers to change, articulating a pathway of change for the targeted behaviour change, and selecting implementation strategy components to overcome identified barriers and enhance enablers of change. ^4^^,^^5^^,^^10^^,^^11^ For example, implementation researchers often select a TMF to inform interview guide development in studies assessing barriers and enablers to healthcare professional adherence to an evidence-based clinical practice guideline. ^8^ Results can be used to inform stakeholders of areas necessitating intervention to enhance implementation outcomes. Table 1 presents French *et al*.,’s ^11^ approach for the development of a theory-informed implementation strategy.

**Table 1.** Suggested steps for the development of a theory informed implementation strategy. Adapted from French *et al.* ^11^ and Wolfenden *et al.* ^4^

Steps	Description
1	Identify who (e.g., individuals or professional groups) needs to do what differently, when and in what context, for implementation to be improved.
2	Using informal and formal theory and frameworks, identify barriers and enablers that need to be resolved, and articulate a pathway of change for the targeted behaviour change to occur. A variety of research methods should be used to support the development of the change pathway (programme theory).
3	Select implementation strategy components (behaviour change techniques, modes of delivery) that might be effective, locally relevant, acceptable, and feasible to overcome identified barriers and enhance enablers to change. Selection of strategies should be based on matrices recommended by determinant frameworks, empirical evidence, and engagement with end users.
4	Decide how implementation can be robustly and feasibly measured, including factors on the hypothesised causal pathway (mediators) and appropriate implementation outcomes.

However, the sheer number of TMFs relevant to implementation strategies is staggering and they are not one-size-fits-all; several complexities must be considered. ^9^^,^^12^ TMFs have different purposes, operate at different levels (e.g., individual, organization, community, system), contain constructs ranging from broad to operational, and the relationships between their constructs can vary (e.g., linear, cyclical, feedback) or be absent. ^13^^,^^14^ TMFs vary in the extent to which there are methods for operationalizing them; some TMFs are associated with a wide range of resources that can be useful to guide implementation strategy design. Finally, the supporting evidence varies; some theories emerged from decades of research in psychology, while this is not the case for more recent TMFs. ^13^^,^^14^ To help clarify TMF distinctions, Nilsen ^8^ proposed a taxonomy identifying five categories of TMFs: process models, determinant frameworks, classic theories, implementation theories, and evaluation frameworks (see Table 2). Three types of TMFs are particularly useful for informing implementation strategy design. Determinant frameworks consolidate several theories and describe multilevel factors that are theoretically and/or empirically linked to implementation outcomes. Classic theories and implementation theories describe precise mechanisms of behaviour and behaviour change that can be targeted by implementation strategy components. These theories often describe how change in the behaviour of those involved in an implementation process (e.g., healthcare professionals, patients) is anticipated to occur. ^4^^,^^8^

**Table 2.** Five categories of theories, models and frameworks used in implementation science. Adapted from Nilsen ^8^ and Wolfenden *et al.* ^4^

Theory, model or framework type	Description
Process models (e.g., Knowledge-to-Action Cycle)	• Models that specify steps (stages, phases) in the process of translating research into practice, including the implementation and use of research.
Determinant frameworks	• Frameworks often developed through the consolidation of constructs from a range of theories and aiming to understand and explain factors that could influence implementation. • They typically do not describe mechanisms for change.
Classic theories	• Theories originating from disciplines such as psychology that help understand or explain individual, group, or organizational behaviour. • They describe precise mechanisms of behaviour change.
Implementation theories	• Theories developed (or adapted classic theories) specifically to understand, explain, and inform implementation. • They describe precise mechanisms of change for one or more aspect of implementation.
Evaluation frameworks	• Frameworks that specify aspects of implementation that could be evaluated to determine implementation success.

While there is a wide range of TMFs available to inform implementation strategy design, researchers have little guidance on the desirable attributes of TMFs for this purpose. Furthermore, there is little information on how to select TMFs for use in their specific context. Several papers have reported broad taxonomies intended to elucidate and identify attributes on which to select TMFs for use in implementation research, but none, to our knowledge, specifically focus on implementation strategy design. Tabak *et al*., ^13^ sought to classify, organize and synthesize models aiming to support dissemination and implementation and offered guidance on how to select a model to inform study design and execution. Davis *et al*., ^15^ developed a list of criteria to appraise the quality of theories of behaviour and behaviour change. Birken *et al*., ^16^^,^^17^ identified the criteria used by implementation scientists to select TMFs and developed a tool to help scientists and practitioners select appropriate TMFs to guide their implementation initiatives. Similar work was undertaken by Lynch *et al*., ^18^ who developed a pragmatic guide for TMF selection. More recently, Strifler *et al*., ^12^ conducted a qualitative study that aimed to explore barriers and facilitators to identifying and selecting TMFs.

**Figure 1.**
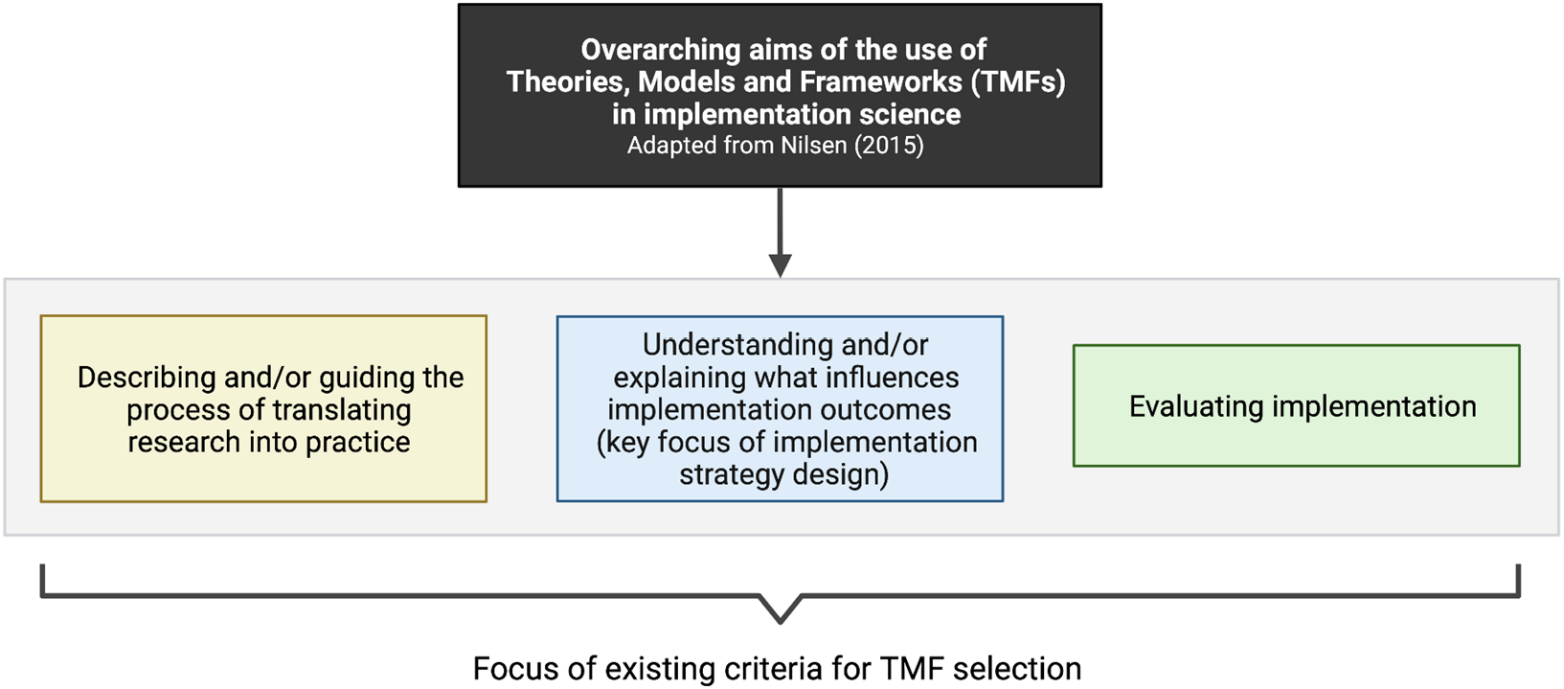
Focus of existing criteria for TMF selection; the criteria are not differentiated in function of the study purpose. The blue box corresponds to the use of TMFs for implementation strategy design.

Despite the breadth of work, current papers discussing TMF selection do not differentiate criteria as a function of how implementation scientists and practitioners intend to use them. ^16^^,^^17^ As discussed by Nilsen, ^8^^,^^19^ there are different overarching aims of the use of TMFs in implementation science, but the selection criteria have not yet been differentiated by function of their intended use ( Figure 1). Criteria currently available remain the same whether implementation scientists and practitioners intend to use TMFs for guiding the process of translating research into practice, understanding what influences implementation outcomes (to guide implementation strategy design), or evaluating implementation.

We posit that classifications of attributes for TMF selection must be more distinctive and focus on their intended use. In the context of implementation strategy design, some criteria for TMF selection might be more relevant than others. Applicability—i.e., whether a particular method (e.g., interviews, surveys, focus groups) can be used with the TMF—is often critical. Furthermore, the extent to which the TMF provides an explanation of how included constructs influence implementation and/or one another is essential. ^16^^,^^17^ Thus, the lack of clear criteria renders TMF selection, and therefore implementation strategy design, perplexing, particularly for neophyte implementation scientists. ^16^^,^^17^ As implementation strategy design is a key focus of implementation research, we believe it is critical to clarify specifically the selection criteria of TMFs in this context. Therefore, it is of high interest to synthesize the literature reporting on the selection of TMFs for informing implementation strategy design.

## Objective

This scoping review aims to identify literature reporting on the selection of TMFs for informing implementation strategy design in healthcare and understand the suggested use of TMFs in implementation strategy design. We found no similar review after a preliminary search in MEDLINE (Ovid) and the Cochrane Database of Systematic Reviews on 15 ^th^ March 2022. The main review question is the following:

1. What are the desirable attributes/selection criteria of TMFs for implementation strategy design in healthcare?

In addition, we will explore the purposes and uses of TMFs for informing each step of implementation strategy design, and the suggested research methods (e.g., qualitative interviews) for use in this context.t

## Methods

Scoping reviews are particularly useful to explore, identify, map and discuss characteristics of concepts across a wide range of evidence sources. ^20^ The proposed scoping review will be conducted in accordance with the Joanna Briggs Institute (JBI) methodology for scoping reviews (which expands on the work of Arksey and O’Malley ^21^ and Levac *et al.* ^22^). It emphasizes appropriateness, feasibility, and meaningfulness of the literature and includes nine sequential steps. First, defining the scoping review objective(s) and question(s). Second, developing and aligning the inclusion criteria with the objective(s) and question(s). Third, specifying the approach to evidence searching, selection, data extraction, and presentation. The next four steps involve searching for, selecting, extracting and analysing the evidence. The eighth is the presentation of the results, while the ninth and last step is summarizing the evidence, making conclusions and noting implications of the findings. ^20^

### Eligibility criteria

**Figure 2.**
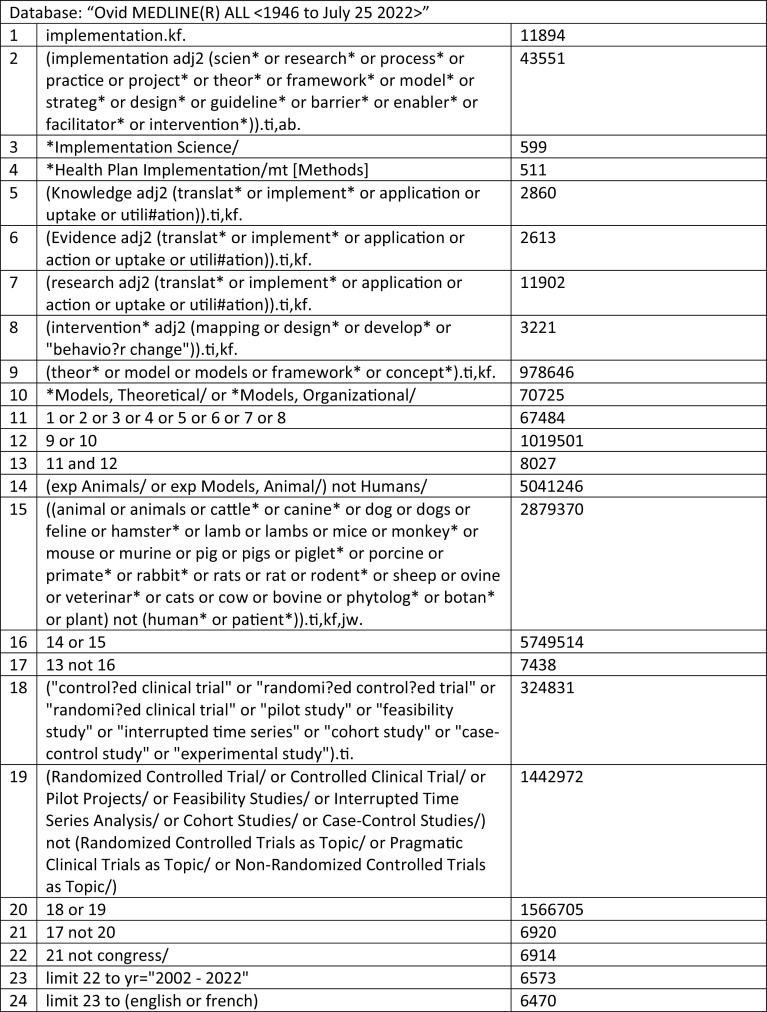
Search strategy.

The eligibility criteria are presented in Table 3 and described in more detail below. We will include literature reporting on a) the desirable attributes or selection criteria of TMFs for informing implementation strategy design and/or on b) the use of TMFs in implementation strategy design (concept) within any healthcare setting (context) ( Figure 2). We define ‘implementation strategy design’ as all steps described in Table 1, including a) the identification of which stakeholders need to do what differently, b) the identification of the barriers and enablers that need to be resolved, c) the selection of implementation strategy components and the identification of the hypothesized casual pathway (mediators), and c) the selection of appropriate implementation outcomes. ^11^ We anticipate that the primary types of records will be methodological papers and expert opinion papers. In addition, systematic reviews that meet the inclusion criteria will also be considered, depending on the research question. Primary studies will be included only if they research the use of TMFs in implementation strategy design; studies aiming to design and evaluate implementation strategies will be excluded. Furthermore, we will consider other types of records if these meet the eligibility criteria regarding the concept and context of interest. After a preliminary search in the grey literature, we concluded that it does not provide a substantial addition to the academic literature. Thus, we decided to not search the grey literature for low relevance and feasibility reasons.

**Table 3.** Eligibility criteria.

Criterion	Inclusion criteria	Exclusion criteria
***Concept***	• We will include papers reporting on the desirable attributes and/or selection criteria of theories, models and frameworks (TMFs) for implementation research • We will include studies researching/investigating the use of TMFs in implementation strategy design (i.e., papers that go beyond a superficial level and provide enough detail to answer the review question)	• We will exclude papers that do not discuss implementation TMFs, their selection criteria/attributes or their use in implementation strategy design • We will exclude studies aiming to design and evaluate specific implementation strategies
***Context***	• We will include papers reporting on the review concept in any healthcare setting/context	• We will exclude papers unrelated to healthcare settings (e.g., work settings, school settings)
***Language***	• We will include papers published in English and French	• We will exclude papers published in any other language
***Time period***	• We will include papers published from 1 ^st^ January 2002 and onward	• We will exclude papers published on or before 31 ^st^ December 2001
***Types of sources***	• We will include review, discussion, methods, and opinion papers as well as editorials focusing on desirable attributes and/or selection criteria of theories, models and frameworks (TMFs) for implementation research • We will also consider primary studies and study protocols researching/investigating the use of TMFs in implementation strategy design for inclusion	• We will exclude conference abstracts and non-peer reviewed sources (e.g., blogs) • We will exclude randomized controlled trials
***Geographic***	• We will include papers originating from all countries	• None

### Literature search

***Information sources***

The bibliographical databases to be searched include MEDLINE (Ovid), CINAHL (EBSCO), and Embase (Ovid). Additionally, we will hand-search relevant journals to identify additional records. Examples of journals may include: *Implementation Science*, *Implementation Science Communications*, *BMC Health Services Research*, *Implementation Research and Practice*, and *Health Research Policy and Systems.* We will screen the reference list of included records to identify additional records. Finally, we will identify a limited number of ‘core papers’ (e.g., 10) and perform a citation search.

***Search strategy***

The search strategy will aim to locate published papers in peer-reviewed journals. An initial limited search of MEDLINE and CINAHL was undertaken in April 2022 to identify articles on the topic. The text words contained in the titles and abstracts of relevant articles, and the index terms used to describe the articles, were used to develop and refine a full search strategy for MEDLINE (see Figure 1). We also adapted elements from the search strategies of three recent reviews in the field of implementation science, Colquhoun *et al*., ^10^ Walsh-Bailey *et al*., ^14^ and Esmail *et al*., ^9^ to fit our objectives. The full search strategy, including all identified keywords and index terms, will be adapted for CINAHL and Embase. The reference list of all included sources of evidence will be screened for additional papers. We will include papers published in English and French only. We will limit the search to 2002–2022 as implementation research has developed drastically over the last two decades. Limiting our search will ensure that information is relevant for use today.

### Source of evidence selection

Following the search, all identified citations will be collated and duplicates removed. Following a pilot test, titles and abstracts will be screened by two independent reviewers for assessment against the inclusion criteria for the review. The full text of selected citations will be assessed in detail against the inclusion criteria by two independent reviewers. Reasons for exclusion of sources of evidence during the full text review that do not meet the inclusion criteria will be recorded and reported in the scoping review. Any disagreements that arise between the reviewers at each stage of the selection process will be resolved through discussion, or with an additional reviewer. The results of the search and the study inclusion process will be reported in full in the final scoping review and presented in a Preferred Reporting Items for Systematic Reviews and Meta-analyses extension for scoping review (PRISMA-ScR) flow diagram. ^23^

### Data extraction

Data will be extracted from included records by two independent reviewers using a data extraction tool developed specifically for this review (see Table 4). The data extracted will include specific details about the concept, context, study methods and key findings relevant to the review question. Using the extraction tool, we will extract the following data from included records:

1. *Descriptive data*: year of publication, first author’s academic discipline, country of origin, article type (e.g., study, editorial, review, opinion paper) and aim.
2. *Methodological data*: study design (if applicable), population and sample size (if applicable), study setting, data collection and analysis methods (if applicable).
3. Data on TMFs:
  a. Authors’ rationale regarding the purpose of TMFs:
    • Generally, in implementation research and practice
    • Specifically for informing implementation strategy design
  b. Authors’ rationale regarding the desirable attributes, selection criteria and selection process of TMFs:
    • Generally, in implementation research and practice
    • Specifically for informing each of the four main steps of implementation strategy design: 1) identifying who needs to do what, differently; 2) identifying barriers and enablers and articulate a pathway of change; 3) selecting implementation strategy components; and 4) deciding how change in implementation will be measured.
  c. Authors’ rationale regarding how TMFs should be used for implementation strategy design, according to the different types of theory use specified in the Theory Coding Scheme. ^24^
  d. The research methods (e.g., intervention mapping) that should be used to incorporate theory in implementation strategy design
4. *Results data:* reported results according to study outcomes (if applicable).

**Table 4.** Data extraction tool.

**General information**	
1. **Study ID *(surname of first author and year first full report of study was published e.g. Smith 2001)***	
2. **Report IDs of other reports of this study *(e.g. duplicate publications, follow-up studies)***	
3. **General notes**	
4. **Date form completed *(dd/mm/yyyy)***	
5. **Name/ID of person extracting data**	
6. **Report title *(title of paper/abstract/report that data are extracted from)***	
7. **Report ID *(if there are multiple reports of this study)***	
8. **Reference details**	
9. **Report author contact details**	
10. **Publication type *(e.g. full report, abstract, letter)***	
11. **Publication aim**	
12. **First author’s academic discipline**	
13. **First author’s country of origin**	
**Study characteristics *(if applicable)***	
14. **Design**	
15. **Population and sample characteristics**	
16. **Setting**	
17. **Types of intervention**	
18. **Types of outcome measures**	
19. **Data collection methods**	
20. **Data analysis methods**	
**Data on the desirable attributes, selection criteria and use of theories, models and frameworks (TMFs)**	
21. **Rationale regarding the purpose of TMFs generally, in implementation research and practice;**	
22. **Rationale regarding the purpose of TMFs specifically for informing implementation strategy design;**	
23. **Rationale regarding the selection process of TMFs;**	
24. **Desirable attributes/selection criteria of TMFs generally, in implementation research and practice;**	
25. **Desirable attributes/selection criteria of TMFs** **for informing the first step of implementation strategy design: 1) identifying who needs to do what, differently;**	
26. **Desirable attributes/selection criteria of TMFs** **for informing the second step of implementation strategy design: 2) identifying barriers and enablers and articulate a pathway of change;**	
27. **Desirable attributes/selection criteria of TMFs** **for informing the third step of implementation strategy design: 3) selecting implementation strategy components**	
28. **Desirable attributes/selection criteria of TMFs** **for informing the fourth step of implementation strategy design: 4) deciding how change in implementation will be measured;**	
29. **Rationale regarding how TMFs should be used for implementation strategy design:**	
a. Suggested use of TMFs to select recipients for the intervention and define the target behaviour? *If yes, paste relevant paper section.*	
b. Suggested use of TMF for selecting the theoretical constructs that the study intervention is hypothesized to change? *If yes, paste relevant paper section.*	
c. Suggested use of a single or a combination of TMFs? *If yes, paste relevant paper section.*	
d. Suggested use of TMFs to select/develop intervention techniques? *If yes, paste relevant paper section.*	
e. Suggested use of TMFs to tailor intervention techniques? *If yes, paste relevant paper section.*	
f. Suggested use of TMFs to link intervention techniques to theory-relevant constructs or predictors, and vice-versa? *If yes, paste relevant paper section.*	
g. Suggested use of TMFs to specify which theory-relevant constructs/predictors will be measured? *If yes, paste relevant paper section.*	
30. **Which research methods (e.g., intervention mapping) should be used?**	
**Study results (if applicable)**	
31. **Main results**	
**Other information**	
32. **Key conclusions of study authors**	
33. **References to other relevant studies**	

Notes on using a data extraction form: 1) Be consistent in the order and style you use to describe the information for each included study; 2) Record any missing information as unclear or not described, to make it clear that the information was not found in the study report(s), not that you forgot to extract it; and 3) Include any instructions and decision rules on the data collection form, or in an accompanying document. It is important to practice using the form and give training to any other authors using the form.

Two independent reviewers will pilot the form by extracting data from five publications and the tool will be revised iteratively. Modifications will be detailed in the scoping review. Any disagreements that arise between the reviewers will be resolved through discussion, or with an additional reviewer on our research team. If appropriate, authors of papers will be contacted to request missing or additional data, where required.

### Data analysis and presentation

An inductive approach will be used to categorize the desirable attributes and selection criteria of TMFs by two researchers independently. This process will be iterative as each researcher will continue categorization until distinct groups are created. After categorizing, the two researchers will compare groupings. Discrepancies in the categories will be resolved in group discussions among the research team. The final categories will be summarized into attribute themes. The entire research team will review final attribute themes, and revisions will be made until all team members agree. Findings will be presented as a list of attributes on which to base TMF selection for guiding implementation strategy design. Descriptive data regarding the purpose, different types of TMF use and research methods in implementation strategy design will be synthesised graphically and in table format. Data will also be presented narratively.

### Future direction

Results of this scoping review can be used to develop a taxonomy based on attributes and criteria specific to TMF selection for implementation strategy design, a key focus of implementation research and practice. Using the results of this scoping review, we plan to classify existing TMFs according to the attributes identified. This will result in additional publications. We also plan on creating an accessible, online tool with which researchers can identify TMFs for use in implementation strategy design. Specifically, the tool will allow researchers to select their intended use for a TMF in implementation strategy design and provide TMFs that match the identified desired traits.

## Data availability

No data are associated with this article.

## References

[1] Proctor EK Powell BJ McMillen JC : Implementation strategies: recommendations for specifying and reporting. *Implement. Sci.* 2013;8(1):139. 10.1186/1748-5908-8-139 24289295 PMC3882890

[2] Powell BJ Beidas RS Lewis CC : Methods to improve the selection and tailoring of implementation strategies. *J. Behav. Health Serv. Res.* 2017;44:177–194. 10.1007/s11414-015-9475-6 26289563 PMC4761530

[3] Powell BJ Waltz TJ Chinman MJ : A refined compilation of implementation strategies: results from the Expert Recommendations for Implementing Change (ERIC) project. *Implement. Sci.* 2015;10(1):1–14. 25889199 10.1186/s13012-015-0209-1 PMC4328074

[4] Wolfenden L Foy R Presseau J : Designing and undertaking randomised implementation trials: guide for researchers. *BMJ.* 2021;372. 10.1136/bmj.m3721 33461967 PMC7812444

[5] Davidoff F Dixon-Woods M Leviton L : Demystifying theory and its use in improvement. *BMJ Qual. Saf.* 2015;24:228–238. 10.1136/bmjqs-2014-003627 25616279 PMC4345989

[6] Eccles M : The Improved Clinical Effectiveness through Behavioural Research Group (ICEBeRG). Designing theoretically-informed implementation interventions. *Implement. Sci.* 2006;1. 10.1186/1748-5908-1-4 16722571 PMC1436012

[7] Eccles M Grimshaw J Walker A : Changing the behavior of healthcare professionals: the use of theory in promoting the uptake of research findings. *J. Clin. Epidemiol.* 2005;58(2):107–112. 10.1016/j.jclinepi.2004.09.002 15680740

[8] Nilsen P : Making sense of implementation theories, models and frameworks. *Implement. Sci.* 2015;10(53):13–53. 10.1186/s13012-015-0242-0 25895742 PMC4406164

[9] Esmail R Hanson HM Holroyd-Leduc J : A scoping review of full-spectrum knowledge translation theories, models, and frameworks. *Implement. Sci.* 2020;15(1):11–14. 10.1186/s13012-020-0964-5 32059738 PMC7023795

[10] Colquhoun HL Squires JE Kolehmainen N : Methods for designing interventions to change healthcare professionals’ behaviour: a systematic review. *Implement. Sci.* 2017;12(30):30. 10.1186/s13012-017-0560-5 28259168 PMC5336662

[11] French SD Green SE O’Connor DA : Developing theory-informed behaviour change interventions to implement evidence into practice: a systematic approach using the Theoretical Domains Framework. *Implement. Sci.* 2012;7(1):38. 10.1186/1748-5908-7-38 22531013 PMC3443064

[12] Strifler L Barnsley JM Hillmer M : Identifying and selecting implementation theories, models and frameworks: a qualitative study to inform the development of a decision support tool. *BMC Med. Inform. Decis. Mak.* 2020;20(1):12–91. 10.1186/s12911-020-01128-8 32408909 PMC7227323

[13] Tabak RG Khoong EC Chambers DA : Bridging research and practice: models for dissemination and implementation research. *Am. J. Prev. Med.* 2012;43(3):337–350. 10.1016/j.amepre.2012.05.024 22898128 PMC3592983

[14] Walsh-Bailey C Tsai E Tabak RG : A scoping review of de-implementation frameworks and models. *Implement. Sci.* 2021;16(1):100–118. 10.1186/s13012-021-01173-5 34819122 PMC8611904

[15] Davis R Campbell R Hildon Z : Theories of behaviour and behaviour change across the social and behavioural sciences: A scoping review. *Health Psychol. Rev.* 2015;9(3):323–344. 10.1080/17437199.2014.941722 25104107 PMC4566873

[16] Birken SA Powell BJ Shea CM : Criteria for selecting implementation science theories and frameworks: results from an international survey. *Implement. Sci.* 2017;12(1):124–129. 10.1186/s13012-017-0656-y 29084566 PMC5663064

[17] Birken SA Rohweder CL Powell BJ : T-CaST: an implementation theory comparison and selection tool. *Implement. Sci.* 2018;13(1):110–143. 10.1186/s13012-018-0836-4 30466450 PMC6251099

[18] Lynch EA Mudge A Knowles S : “There is nothing so practical as a good theory”: a pragmatic guide for selecting theoretical approaches for implementation projects. *BMC Health Serv. Res.* 2018;18(1):811–857. 10.1186/s12913-018-3671-z 30428882 PMC6236961

[19] Nilsen P Birken SA : *Handbook on Implementation Science.* Edward Elgar Publishing;2020.

[20] Peters MDJ Godfrey C McInerney P : Chapter 11: Scoping reviews (2020 version). Aromataris E Munn Z , editors. *Joanna Briggs Institute (JBI) manual for evidence synthesis.* JBI;2020.

[21] Arksey H O'Malley L : Scoping studies: Towards a methodological framework. *Int. J. Soc. Res. Methodol.* 2005;8(1):19–32. 10.1080/1364557032000119616

[22] Levac D Colquhoun H O'Brien KK : Scoping studies: Advancing the methodology. *Implement. Sci.* 2010;5(1):69. 10.1186/1748-5908-5-69 20854677 PMC2954944

[23] Tricco AC Lillie E Zarin W : PRISMA extension for scoping reviews (PRISMA-ScR): checklist and explanation. *Ann. Intern. Med.* 2018;169(7):467–473. 10.7326/M18-0850 30178033

[24] Michie S Prestwich A : Are interventions theory based? Development of a theory coding scheme. *Health Psychol.* 2010;29(1):1–8. 10.1037/a0016939 20063930

