# Supplementary material for: Desirable attributes of theories, models, and frameworks for implementation strategy design in healthcare: a scoping review protocol
Source: F1000Res. 2022 Sep 6;11:1003. [Version 1] doi: 10.12688/f1000research.124821.1 (PMC10382787; doi:10.12688/f1000research.124821.1)
Supplement: Peer review history

# Peer review: Reviewer response for version 1

**DOI:** 10.5256/f1000research.137056.r187545

**Published:** 2023-07-28

**Authors:** Lauren Connell Bohlen

**Affiliations:** 1 Center for Health Promotion and Health Equity, Brown University School of Public Health, Providence, RI, USA

The protocol for a scoping review to investigate the use of theories, models, and frameworks in the development of implementation strategies is well written. My one reservation with the protocol is that as worded, the research question to identify 'desirable attributes' is not clearly operationalized. The authors should specify how the data extracted, and the analysis plan will lead to the identification of a 'desirable' attribute. Additionally, it is unclear whether all of the items from the theory coding scheme will be extracted, or whether only some will be extracted and in a modified way (e.g., item 3c).

Is the study design appropriate for the research question?

Yes

Is the rationale for, and objectives of, the study clearly described?

Partly

Are sufficient details of the methods provided to allow replication by others?

Partly

Are the datasets clearly presented in a useable and accessible format?

Not applicable

Reviewer Expertise:

Behavioral science, Behavior change theories and frameworks, Implementation science

I confirm that I have read this submission and believe that I have an appropriate level of expertise to confirm that it is of an acceptable scientific standard, however I have significant reservations, as outlined above.

# Peer review: Reviewer response for version 1

**DOI:** 10.5256/f1000research.137056.r187547

**Published:** 2023-07-21

**Authors:** Sanne Peters

**Affiliations:** 1 School of Health Sciences, Faculty of Medicine, Dentistry and Health Sciences, The University of Melbourne, Melbourne, Victoria, Australia

This paper is a protocol for a scoping review that aims to 1) identify the literature reporting on the selection of theoretical approaches for informing implementation strategy design in healthcare and 2) understand the suggested use of these approaches in implementation strategy design. The rationale for this review is clearly described and the methods are transparent and appropriate for the research questions. I believe that this will be a very informative review.

 I have one suggestion for the authors to consider. I think that it is a great idea to synthesize desirable attributes/selection criteria. I am wondering whether you can give recommendations regarding prioritisation of selection criteria, based on your review findings and/or authors' expertise? For example, do we need to give more "weight" to one or two selection criteria and why? Can you order the selection criteria based on importance/relevance (from most desirable to least desirable) as described in the literature? Or does the order of selection criteria depend on the local context and should researchers make their own selection of X number of criteria and decide their order? How many criteria would you suggest? I wonder if you can find that kind of information in the literature. I have used multiple selection criteria, for other purposes, in the past and I feel that some criteria are more important than others. It would be informative to give guidance on how to deal with this. This is just a suggestion and the authors are free to ignore it.

 I believe that this is a very useful scoping review. I am very much looking forward to reading the review findings and wish the author team the best of luck.

Is the study design appropriate for the research question?

Yes

Is the rationale for, and objectives of, the study clearly described?

Yes

Are sufficient details of the methods provided to allow replication by others?

Yes

Are the datasets clearly presented in a useable and accessible format?

Not applicable

Reviewer Expertise:

Implementation Science, Knowledge Translation and Medical Education.

I confirm that I have read this submission and believe that I have an appropriate level of expertise to confirm that it is of an acceptable scientific standard.

# Peer review: Reviewer response for version 1

**DOI:** 10.5256/f1000research.137056.r179778

**Published:** 2023-07-06

**Authors:** Thomas J. Waltz

**Affiliations:** 1 Department of Psychology, Eastern Michigan University, Ypsilanti, Michigan, USA

The authors describe a study protocol for a scoping review of studies focused on the selection of theories, models, and frameworks used to inform any of the four steps involved in developing a theory-informed selection of implementation strategies. The aim is to be able to characterize the desirable attributes/selection criteria of theories, models, and frameworks aiding implementation strategy design with a focus on applications to healthcare. The rationale for the scoping review is clearly described and justified. The details of the search strategy are transparently reported and the data extraction plan is consistent with the aim of the project.

 My substantive comments focus on the review’s inclusion/exclusion criteria.

 1) In Table 3, the Context domain is over-exclusionary. Implementation of health and mental health services in school settings is a robust area of implementation research. The same is true for health and nutrition programs within childcare settings. I suggest the inclusion criteria for context be amended to read something like: We will include papers reporting on the review concept in any healthcare setting/context inclusive of schools and childcare settings where the implementation research focuses on the provision of health-related care in that setting.

 The search strategy in Figure 2 suggests that this suggestion is feasible since key terms related to “school” were not explicit exclusions in the search strategy. If this friendly amendment to the inclusion/exclusion criteria is viewed as impractical, the authors should provide additional justification for why the exclusion of health-related care in school and childcare settings is necessary.

 2) It is unclear whether studies on policy implementation would be included if the subject of the policy area involves healthcare. Please clarify.

 Minor comment.

 3) There is a typo on page 5: the last line before the Methods header has an extra “t” following the end of the sentence.

Is the study design appropriate for the research question?

Yes

Is the rationale for, and objectives of, the study clearly described?

Yes

Are sufficient details of the methods provided to allow replication by others?

Yes

Are the datasets clearly presented in a useable and accessible format?

Not applicable

Reviewer Expertise:

Implementation science, clinical psychology, behavioral economics

I confirm that I have read this submission and believe that I have an appropriate level of expertise to confirm that it is of an acceptable scientific standard, however I have significant reservations, as outlined above.
